# Comparison of Targeted and Untargeted Approaches in Breath Analysis for the Discrimination of Lung Cancer from Benign Pulmonary Diseases and Healthy Persons

**DOI:** 10.3390/molecules26092609

**Published:** 2021-04-29

**Authors:** Michalis Koureas, Dimitrios Kalompatsios, Grigoris D. Amoutzias, Christos Hadjichristodoulou, Konstantinos Gourgoulianis, Andreas Tsakalof

**Affiliations:** 1 Department of Hygiene and Epidemiology, University Hospital of Larissa, Faculty of Medicine, University of Thessaly, 22 Papakyriazi Street, 41222 Larissa, Greece; mkoureas@med.uth.gr (M.K.); thejimkal@gmail.com (D.K.); xhatzi@med.uth.gr (C.H.); 2 Bioinformatics Laboratory, Department of Biochemistry and Biotechnology, University of Thessaly, 41500 Larissa, Greece; amoutzias@bio.uth.gr; 3 Respiratory Medicine Department, University Hospital of Larissa, Faculty of Medicine, University of Thessaly, 41110 Larissa, Greece; kgourg@uth.gr; 4 Department of Biochemistry, Faculty of Medicine, University of Thessaly, 41500 Larissa, Greece

**Keywords:** lung cancer, exhaled breath, volatile organic compounds, untargeted analysis, breath analysis, cancer biomarkers, volatolomics

## Abstract

The aim of the present study was to compare the efficiency of targeted and untargeted breath analysis in the discrimination of lung cancer (Ca+) patients from healthy people (HC) and patients with benign pulmonary diseases (Ca−). Exhaled breath samples from 49 Ca+ patients, 36 Ca− patients and 52 healthy controls (HC) were analyzed by an SPME–GC–MS method. Untargeted treatment of the acquired data was performed with the use of the web-based platform XCMS Online combined with manual reprocessing of raw chromatographic data. Machine learning methods were applied to estimate the efficiency of breath analysis in the classification of the participants. Results: Untargeted analysis revealed 29 informative VOCs, from which 17 were identified by mass spectra and retention time/retention index evaluation. The untargeted analysis yielded slightly better results in discriminating Ca+ patients from HC (accuracy: 91.0%, AUC: 0.96 and accuracy 89.1%, AUC: 0.97 for untargeted and targeted analysis, respectively) but significantly improved the efficiency of discrimination between Ca+ and Ca− patients, increasing the accuracy of the classification from 52.9 to 75.3% and the AUC from 0.55 to 0.82. Conclusions: The untargeted breath analysis through the inclusion and utilization of newly identified compounds that were not considered in targeted analysis allowed the discrimination of the Ca+ from Ca− patients, which was not achieved by the targeted approach.

## 1. Introduction

Human breath contains volatile organic compounds (VOCs) either originating from endogenous biochemical processes and thus distinguished as endogenous VOCs or environmental exposures (inhalation, ingestion, dermal absorption) and therefore pertaining to exogeneous VOCs. In case of disease, the biochemical pathways can be dysregulated or altered [1], and this will change the composition of exhaled breath in endogenous VOCs. Moreover, disease can also affect the absorption, distribution metabolism and excretion of the exogenous compounds. These alterations can be detected and used for disease detection and diagnosis. The analysis of exhaled breath is currently an area of intensive research aiming at the development of new non-invasive tests for preliminary screening and diagnosis of various pathological conditions. Particular attention is given to cancer, where early diagnosis is critical for successful disease treatment and which today is often diagnosed at late stages, and diagnosis procedures are invasive, time consuming or costly. Mass spectrometry (MS)-based breath analysis for disease diagnosis research is currently the mainstream choice that can be accomplished using two strategies, which are classified as targeted or non-targeted (also referred to as untargeted). The former is based on quantification of an a priori defined set of VOCs known or hypothesized as disease biomarkers and is thus a hypothesis-driven approach. In contrast, the non-targeted strategy is a (qualitative) hypothesis-generating approach that investigates the whole VOC profile in a breath sample without any a priori information about the chemical composition of the sample and aims to identify a maximum number of VOCs. By non-targeted breath analysis, novel biomarkers and disturbed metabolic pathways can be discovered or characteristic breath VOC profile of the disease can be defined and further used for disease detection and diagnosis. However, the non-targeted approach yields a huge amount of complex data and its application would be impossible without the development of bioinformatics software designed for the treatment and statistical analysis of raw chromatography–mass spectrometry data, and identification of detected unknown compounds. This has been done mostly in the last decade and currently there is a variety of commercial or open source software for the treatment and analysis of chromatography–mass spectrometry data and extraction of the relative biological information [2]. That has given great impetus for the development of non-targeted analysis in metabolomics in general [3] and opens new perspectives in breath research in particular [4]. One of the most widely used metabolomic software is XCMS Online, which is freely available [5].

However, the non-targeted approach has long-standing reproducibility issues [6,7] and is never truly unbiased since the acquired data are significantly affected by experimental design and instrumental parameters. In contrast to the targeted strategy, the lack of absolute quantification makes it difficult to assess variations in metabolite levels between groups, to normalize the acquired data and even to make interlaboratory comparisons of the results [7,8]. These weaknesses of the non-targeted approach are, at the same time, the strengths of the targeted approach and, recently, hybrid approaches bridging them have been developed [8,9]. In this study, we make a retrospective non-targeted analysis of full scan data previously acquired [10] in targeted analysis of the breath samples from lung cancer (Ca+) and benign pulmonary disease (Ca−) patients and healthy controls (HC). The targeted analysis was based on the quantitation of 19 pre-determined VOCs [10]. While Ca+ patients were satisfactorily discriminated from healthy controls, the analysis failed to discriminate Ca+ patients from Ca− patients (without LC but with pathological computed tomography findings). The aim of the present study is to compare the efficiency of the targeted and untargeted approaches in lung cancer discrimination with healthy people and patients with other pulmonary diseases and record the strengths and limitations of each approach on the same raw GC–MS data pool. Additionally, by merging (combining) targeted and untargeted approaches, we sought to improve the discrimination ability of the breath analysis.

## 2. Results

### 2.1. Characteristics of Study Participants

From the 85 patients with pathological computed tomography (CT) findings who underwent bronchoscopy, lung cancer was diagnosed in 49 patients (43 males/6 females). The mean age of Ca+ patients was 71.1 years (SD: 8.2). The majority of LC patients (n = 40) were diagnosed with non-small cell lung carcinoma, while 8 were diagnosed with small cell lung carcinoma (for one patient, the type was not available). Thirty-six patients (30 males/6 females, mean age 66.8 (SD: 10.8)) were not diagnosed with LC by histological/cytological examination. The possible pathological origins for this group include sarcoidosis, hypersensitivity pneumonitis, interstitial lung diseases or pulmonary infections such as tuberculosis. The control group consisted of 52 persons (35 males/17 females) with a mean age of 66.8 (SD: 10.8).

In regard to smoking habit, most of the LC patients (81.6%) were former smokers with a mean time from cessation of 9.4 years, while 12.2% were active smokers and 6.1% reported that they had never smoked. Patients that were not diagnosed with LC had slightly different frequencies of smoking habit, with 55.6% being former smokers (mean time from cessation: 10.6 years), 27.7% being active smokers and 16.7% never smokers. In the HC group, the percentage of active smokers was significantly higher (38.4%), as was the percentage of individuals that had never smoked (28.9%). The percentage of former smokers was 32.7%, with mean time from cessation of 20.1 years. Mean pack/years were 69.43 (SD: 48.47) for the Ca+ group, 48.70 (SD: 35.41) for the Ca− group and 32.74 (SD: 33.39) for the HC group.

Concerning self-reported co-morbidities derived from personal interviews with the use of questionnaires, the most common were hypertension (Ca+ group: 44.9%, Ca− group 47.22%, HC group: 42.31%), diabetes (Ca+ group: 24.49%, Ca− group 27.78%, HC group: 22.45%) and hypercholesterolemia (Ca+ group: 38.78%, Ca− group 30.56%, HC group: 26.53%).

### 2.2. Data Pre-Processing, Selection and Identification of Candidate Features

**Figure 1:**
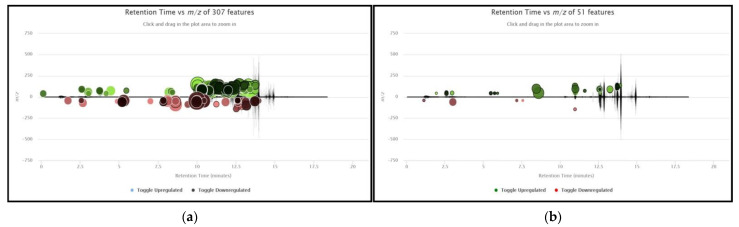
Cloud plots with results of pairwise XCMS analysis between (**a**) Ca+ vs. HC. Detection settings: *p*-value < 0.01, fold change > 2, *m*/*z* range: 0–140, retention range: 0–14 min, max intensity > 10,000 and (**b**) Ca+ vs. Ca− characteristics (ion). Detection settings: *p*-value < 0.05, fold change > 1.1, *m*/*z* range: 0–140, retention range: 0–14 min, max intensity > 10,000.

**Figure 2:**
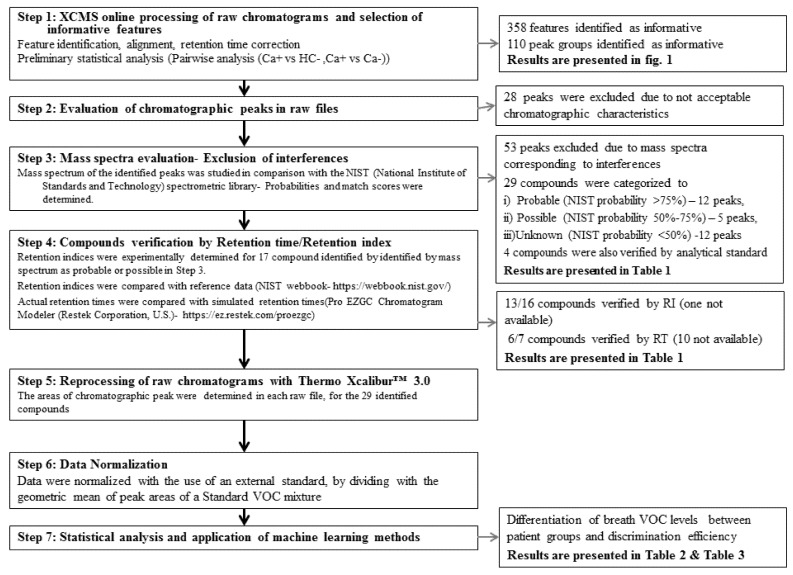
Flow chart of the process applied for selecting, identifying and processing informative compounds.

The processing of raw files with the use of the XCMS Online platform identified 358 informative features (ions) meeting the criteria defined in the Materials and Methods (Section 4.3) after peak identification, alignment, retention time correction and preliminary online statistical analysis. Figure 1 presents the metabolomic cloud plots obtained from XCMS Online, concerning the pairwise analysis of Ca+ vs. HC and Ca+ vs. Ca− groups. Features identified as differentiated between subgroups by XCMS Online were automatically grouped into 110 corresponding chromatographic peaks. These peaks were manually evaluated and verified in the acquired chromatograms. This process resulted in the exclusion of 28 peaks from further analysis due to unacceptable chromatographic characteristics such as low signal to noise ratio and co-elution with other substances. The mass spectra corresponding to the 82 remaining peaks were compared with those stored in the NIST library after subtracting mass spectra corresponding to noise. These procedures lead to the exclusion of additional peaks with spectra indicating silanes and silicon compounds that were considered interferences from SPME fiber, the chromatography column or septum materials. In addition, peaks with mass spectra corresponding to known contaminants from Tedlar^®^ bag materials (phenol, *N,N*-dimethylacetamide) were also excluded [11]. In total, 53 compounds were not considered for further analysis. Thus, the remaining 29 peaks were considered for further investigation. For these, comparisons of mass spectra with those contained in the NIST library identified 12 compounds with a probability higher than 75%. Four monoaromatic compounds (benzene, styrene, ethylbenzene and toluene) were also verified with analytical standards. In addition, seven compounds were verified by retention time (RT) by comparing actual RTs with simulated RTs determined with the use of the Pro EZGC Chromatogram Modeler (Restek Corporation, Bellefonte, PA, USA). For 5 peaks, the NIST probability was 50–75%, indicating a considerable degree of uncertainty in compound identification, while 12 compounds (probability < 50%) were designated as unknowns. Moreover, experimentally determined retention indices (RIs) were compared with those stored in the NIST library. Small deviations were observed (<10%) for most compounds, while the RI values were in agreement with the order of elution of identified VOCs, with the exceptions of propionic acid and methylacetamide. Figure 2 presents the flow chart of the process applied for selecting and identifying informative compounds. In Table 1, the compounds are presented along with NIST probability and spectra match scores, actual and simulated retention times, experimentally determined RIs and RIs derived from the NIST workbook. The 17 identified compounds were further investigated by searching for their presence in the KEGG pathway database [12] and in the scientific literature to determine their putative origins and the involved metabolic pathways. For twelve compounds, no evidence of endogenous origin was found. These include monoaromatic hydrocarbons and furans, which are carcinogens contained in tobacco smoke, and produced by industrial sources and commercial uses, sulfur-containing compounds (methyl propyl sulfide, 1-methylthio-(E)-1-propene) used as flavor agents and contained in garlic and onion and eucalyptol, which is used as an asthma/COPD drug. Eight substances could be of both endogenous and exogenous origin. Most of the identified metabolic pathways concerned the degradation/metabolism of xenobiotic substances such as ethylbenzene, benzene and dimethyacetamide. Propionic acid is involved in multiple pathways of lipid biosynthesis, propanoate metabolism and vitamin K metabolism. P-benzoquinone can be formed from benzene metabolism [13], but also participates in other pathways, and acetic acid is involved in the formation of glycogen, cholesterol synthesis, fatty acid degradation and acetylation of amines [14].

### 2.3. Reprocessing of Raw Chromatographs and Statistical Analysis of Identified/Verified Associations

Following the identification of the compounds, all raw files were reprocessed with Thermo Xcalibur™ software to obtain more valid data. This procedure allowed manual retention time correction, more accurate integration of chromatographic peaks and exclusion of false (noise) peaks. The areas of the chromatographic peaks were determined for each compound in exhaled breath samples but also in ambient air samples. Chromatographic peak areas were normalized with the use of an external standard mixture (see Section 4.4). Regarding ambient air levels, for six out of 29 compounds, the relative levels of ambient air were considered insignificant, for 5 compounds low, for 14 compounds moderate and for 4 compounds high (Table 2). Comparative statistical analysis confirmed the significant difference in breath levels between Ca+ patients and healthy controls for 18 out 29 compounds, while two were found to differ between Ca+ and Ca− patients. Lung cancer patients had significantly elevated levels of ethylbenzene, styrene, toluene, xylene, eucalyptol and four unknown compounds compared to healthy controls. Lower levels were observed for acetaldoxime, methyl propyl sulfide, 1-methylthio-(*E*)-1-propene, propionic acid, methylacetamide and three unknown compounds. Results concerning the comparative analysis of areas of chromatographic peaks between patient groups are summarized in Table 2.

### 2.4. Application of Machine Learning Methods to Estimate the Diagnostic Efficiency of the Breath Analysis

In our previous work, based on 19 selected VOCs, we identified subsets of features (VOCs) that were capable of efficiently discriminating healthy individuals from cancer patients, but not Ca+ from Ca− patients. In this section, we present the results of machine learning methods based on combinations of the 29 features, identified as differentiated between population subgroups by the untargeted approach. When all 29 features were included, correct classification of Ca+ and HC was 86% (AUC: 0.94) (Table 3, Analysis no. 9). After the two steps of feature selection, using a subset of eight features, the correct classification improved to 91% (AUC: 0.96) (Table 3, Analysis no. 10), which was higher than that of targeted analysis. Similarly, discrimination between Ca− patients and HC was also very efficient. The correct classification of datapoints ranged from 90% (AUC: 0.94), when using all 29 features (Table 3, Analysis no. 11), to 94% (AUC: 0.97) after the two steps of feature selection, using a subset of seven compounds (Table 3, Analysis no. 12). Not surprisingly, discrimination between pooled cancer-positive and non-cancer patients (Ca+ and Ca−) and HC was again very efficient. Overall, machine learning models based on compounds identified as differentiated by the untargeted approach achieved a very comparable if not marginally better accuracy than the targeted approach, when trying to discriminate healthy individuals from any of the three types of patients (cancer, non-cancer, pooled).

Subsequently, we tested the potential for discrimination between Ca+ and Ca− patients, with the three machine learning algorithms, by using normalized peak areas of compounds from breath. The set of 29 VOCs was not capable of efficiently discriminating between cancer and non-cancer patients, irrespective of the machine learning algorithm applied. The best-performing algorithm (random forest) correctly predicted only 53% of datapoints (AUC: 0.54) (Table 3, Analysis no. 15) when using all 29 VOCs. However, when two successive steps of feature selection were implemented, the random forest’s accuracy significantly increased to 75% (AUC: 0.82), by using a set of only three metabolites (Table 3, Analysis no. 16). We repeated the analysis to discriminate Ca+ from Ca− patients, by incorporating normalized levels after subtracting ambient air levels, in the hope that removal of any noise from the air would increase the discriminatory power of the random forests. However, the performance did not increase as much as it did when we used only normalized concentrations of breath. More specifically, by using all 29 VOCs, random forests achieved an accuracy of 58% (AUC: 0.54) (Table 3, Analysis no. 17), whereas, after two steps of feature selection, the performance was increased to an accuracy of 72% (AUC: 0.78) by using eight features (Table 3, Analysis no. 18).

We also examined whether the combination of the 19 VOCs measured by the targeted approach together with the 29 VOCs identified as differentiated by the untargeted approach would increase the discriminatory power of the machine learning models in Ca+ vs. Ca− patients. In this set, the concentrations of 19 VOCs in breath were used together with 29 VOCs selected as informative by the untargeted approach. By using all 48 variables, random forests achieved an accuracy of 45% (AUC: 0.44) (Table 3, Analysis no. 19), whereas, after two steps of feature selection, the performance was increased to an accuracy of 73% (AUC: 0.72) (Table 3, Analysis no. 20), using three features (thiophene from the targeted approach and acetaldoxime and N-methyl acetamide from the untargeted approach). Thus, the inclusion of the 19 targeted metabolites did not increase the discriminatory performance of random forests that were based only on targeted metabolites.

Finally, we tested if smoking was a confounding factor for the discrimination (with random forests) of cancer vs. non-cancer patients, using normalized breath measurements of VOCs selected as informative by the untargeted approach. In these analyses, we retained 43 cancer patients and 26 non-cancer patients that never smoked or had quit smoking. The best-performing algorithm (random forest) correctly predicted only 59% of datapoints (AUC: 0.57) when using all 29 untargeted VOCs (Table 3, Analysis no. 21). When we used the three untargeted VOCs that had yielded the best performance in the previous cancer vs. non-cancer patients analysis, random forests of the non-smokers achieved an accuracy of 72.5%, but with a significantly lower AUC of 0.68 (Table 3, Analysis no. 22). Thus, we also performed two rounds of feature selection specifically for the non-smokers and, this time, random forests achieved an accuracy of 77%, with an AUC of 0.85, by using five VOCs (Table 3, Analysis no. 23).

In summary, based on all the above analyses, we conclude that the best-performing algorithm is again random forests, whereas the normalized breath data from the untargeted approach are sufficient to help the algorithm achieve a very high performance, in all comparisons. Furthermore, the two successive rounds of feature selection significantly improved the performance of the random forests, especially in the case of Ca + vs. Ca− patients. This was not possible in a previous study that had used a limited set of 19 selected VOCs. Furthermore, smoking was not a confounding factor for the untargeted analysis, an observation that is in agreement with the results of targeted analysis. It is very clear that the given untargeted approach, in combination with machine learning algorithms and feature selection, identified sets of compounds with sufficient discriminatory power (accuracy of 91–94%) to help us understand if a sample comes from a healthy person or from a person with a pulmonary disease. This was achievable with only seven to nine metabolites. Furthermore, it is also possible to discriminate, with satisfactory accuracy (75–77%), cancer from non-cancer patients, by using only three to five untargeted metabolites.

## 3. Discussion

In this study, we performed analyses based on non-targeted screening of the raw chromatographic data obtained from breath analysis, for three population groups (Ca+, Ca− and HC) and compared the discriminatory power of this approach to that achieved by targeted analysis. In the targeted analysis, 19 pre-selected compounds were measured, which were selected based on literature indicating that they might be potential biomarkers of lung cancer. Seven of these pre-selected compounds were found to differ significantly between Ca+ and HC, and between pooled patient (Ca+ and Ca−) and HC groups, and none differed significantly between Ca+ and Ca− groups [10].

The non-targeted analysis was performed with the use of the XCMS Online data processing platform combined with manual processing of the raw chromatograms to select the informative compounds and develop a dataset containing the areas of chromatographic peaks of differentiated compounds. Processing of the raw files with XCMS Online was conducted to determine the subset of chromatographic peaks and corresponding ions (*m*/*z*) to focus on, and narrow the investigated peaks to those only identified as significantly differentiated between population subgroups (Figure 2: Step 1). Next, we manually cross-checked (Figure 2: Step 2) and reprocessed (Figure 2: Step 5) the identified peaks in the raw data, by integrating extracted ion chromatograms (EICs). This task was performed to confirm and, when necessary, correct the results obtained from automated online data processing, and increase the reliability of the developed dataset, before proceeding to statistical analyses and the application of machine learning methods. We considered this stage necessary since peak misalignment or identification of “false peaks” by preprocessing software has been reported as a potential limitation of this approach due to the variance and complexity of raw chromatograms [15,16,17]. Indeed, a number of peaks identified by XCMS as informative could not be satisfactorily processed in the raw chromatograms and had to be excluded from the analysis, due to noise interferences or co-elution issues. It was interesting that two compounds (1-propanol and 2-propanol) identified as differentiated between population groups by the targeted approach were filtered out by the selection criteria applied in the untargeted workflow. By searching for 2-propanol and 1-propanol in the XCMS results, we observed that the corresponding peaks were correctly identified and their levels were found to differ between Ca+ and HC, while fold changes in LC patients were in agreement with those observed when concentrations determined by calibration curves (targeted analysis) were compared. However, the level of statistical significance of non-normalized values (determined by a *t*-test) was 0.0185 for 2-propanol and 0.0198 for 1 propanol, which was marginally higher than the selection criterion (*p* < 0.01) set for Ca+ vs. Ca− pairwise (online automated) analysis. It should also be mentioned that the *t*-test is not the appropriate significance criterion for non-normally distributed data.

It is also noteworthy that 53 compounds identified as informative by the analysis with XCMS Online were at a later stage excluded as they corresponded to silicon-based compounds and presumably derived from the SPME fiber and chromatographic column bleed (Figure 2: Step 3). The vast majority of these compounds were selected based on the Ca+ vs. HC pairwise analysis and the associations can be attributed to different experimental conditions during the time periods of the collection and analysis of the population subgroups. It is therefore assumed that these compounds were selected due to systematic variations in experimental conditions. This effect is often corrected through normalization processes where signal intensity is adjusted by the total intensity, the highest value or by an external or internal standard [18]. In untargeted metabolomics, the use of pooled samples as external standards is often applied [19] but this practice would be extremely complicated in exhaled air samples. In the present study, external standard normalization was conducted by incorporating spiked standard mixtures with known concentrations that were used in targeted analysis (Figure 2: Step 6). Moreover, after manual processing of the detected peaks and external standard normalization, a few associations that were determined as significant from XCMS Online analysis were not confirmed by offline statistical analysis of reprocessed data.

Some of the identified compounds have been reported previously to differ in the breath of LC patients and other pulmonary diseases. In particular, monoaromatics are reported by numerous publications. A very recent review by Ratiu identified 21 aromatic hydrocarbons differentiated in lung cancer [20]. Furans, such as 3-methylfuran and 2,5-dimethylfuran, have also been identified by previous studies but these compounds are considered biomarkers of both active and passive exposure to tobacco smoke [21]. Allyl methyl sulfide and methyl propyl sulfide (an isomer of 1-methylthio-(*E*)-1-propene), which were found in lower levels in LC patients, are known to suppress the proliferation of human lung tumor cells and possess anti-carcinogenic properties [22,23]. Moreover, similar structures, such as dimethyl sulfide and methionol, are involved in the metabolism of methionine [24]. Differences in the exhaled breath levels of acetic acid and propionic acid have also been reported by previous studies, albeit less frequently [25,26]. Exhaled p-benzoquinone has been proposed as a marker of malignant pleural mesothelioma [27]. For other identified substances (N-2-Aminoethyl acetamide, 1 methoxy propanol, methylacetamide, acetaldoxime, eucalyptol), we did not find any references in the scientific literature concerning the potential association of the exhaled breath concentrations with lung cancer. It should be noted that for some compounds (e.g., propionic acid, acetic acid), we report lower levels in the exhaled breath of LC patients, a finding which apparently contradicts existing evidence. The lack of reproducibility between independent research groups is a known obstacle in breath research. It should also be mentioned that for a few compounds, the identification is questionable. This statement is based on the observation that deviations in RTs and RIs (Figure 2: Step 4) for these compounds do not follow the trend established by known compounds. These include propionic acid, methylacetamide, acetaldoxime and 1-methxy-propanol. The utilization of retention indices in compound identification confirmation through the comparison with available retention data can be of great importance, especially when mass spectral matches are derived from multiple candidate compounds with similar spectra (e.g., isomer compounds) [28]. In our investigation, the use of RIs assisted in the confirmation of mass spectra matches and in distinguishing which isomer compound corresponds to the chromatographic peak (1-methylthio-(E)-1-propene, p-xylene). The small deviations between calculated and library-derived RIs were expected since RIs were experimentally determined with a DB-624 column (6% cyanopropyl/phenyl, 94% polydimethylsiloxane (PDMS)) and retrieved RIs were related to a 100% PDMS column. Naturally, the RI is dependent on the kind of stationary phase and different stationary phases give rise to different RIs of the same compound. However, the same trend in the abovementioned deviation was observed in the vast majority of the identified compounds. The combination of mass spectra and RI data has been proposed in both targeted and untargeted GC–MS data processing protocols [29].

By searching for the identified compounds in metabolic pathway databases and in the scientific literature, we found no direct evidence linking these VOCs to biochemical alterations that occur in cancer and therefore the biochemical interpretation of the results is not straightforward. While instrumental techniques, sampling methods and informatics approaches for studying diseases through the analysis of exhaled breath are constantly evolving [30,31,32], it is critical for future research to advance the knowledge concerning the understanding of underlying mechanisms that result in alteration of VOC breath composition. Current scientific knowledge provides some evidence and hypotheses concerning the biochemical background of endogenous VOCs [33], but the origin of the majority of these compounds is largely uncertain. Further research on endogenous products is of great importance not only for diagnostic purposes but also for targeting treatment [34].

It is evident that most of the compounds identified as differentiated in population groups in the present study are of exogenous origin or are produced endogenously during the metabolism of exogenous compounds. This observation enhances the findings of our previous publication, where it was hypothesized that alterations in pulmonary function and in the metabolism and excretion of exogenous compounds in disease can have an effect on the concentrations measured in exhaled breath. This hypothesis is also supported by several clinical tests and recent research that use exogenous VOCs (EVOCs) as probes to “measure the activity of metabolic enzymes in vivo, as well as the function of organs, through breath analysis” [35]. Future research should further elucidate the potential of the administration of harmless exogenous compounds as probes to study diseases.

In accordance with acquired data, the discrimination of LC patients from patients with abnormal CT findings was substantially increased by the untargeted approach and subsequent feature selection/machine learning in comparison to a previously conducted targeted approach. The correct classification was 75–77% for Ca+ vs. Ca− in the untargeted analysis compared to approximately 50% in the targeted analysis. Additionally, we report 91% accuracy for the discrimination of LC patients from healthy controls based on the investigation of 29 VOCs selected as informative by a non-targeted approach. The discriminatory power was slightly increased compared to the targeted analysis focusing on the quantification of a set 19 pre-determined VOCs. Although the targeted approach has the advantage of the absolute determination of VOC levels and is less prone to biases, untargeted screening allowed us to detect new distinctive features and incorporate a larger compound set into the classification analysis, thus resulting in better discrimination. Previous studies investigating VOC profiles by gas chromatography–mass spectrometry also reported high discriminatory power in distinguishing LC patients from healthy controls [36,37,38,39,40,41,42,43,44,45,46,47]. However, the major concerns are the limited reproducibility regarding the compounds identified by different research groups and the uncertainties regarding the origins of VOCs that differentiate lung cancer. The lower discriminant power between Ca+ and Ca− patients underlines the importance of evaluating the interference of other pulmonary diseases in the identification of LC biomarkers [46,47]. The combination of the datasets developed by the targeted and untargeted approaches did not significantly improve the discrimination, an observation that underlines that the information provided by targeted analysis is contained to a large extent in the data obtained by the untargeted approach. Untargeted VOC screening detected four (toluene, benzene, styrene, ethylbenzene) out of seven compounds that were found to differ significantly in targeted analysis, and exploited numerous features that could not be identified by the targeted approach. In agreement with targeted analysis, incorporating breath subtracts (ambient air was subtracted from breath measurements) slightly decreased the discriminatory power of the analysis. This can be explained by the fact that for some VOCs with high concentrations in ambient air, the information contained in breath measurements was not exploited. Including breath substrate (also referred to as alveolar gradient) in the analysis is a double-edged decision. On the one hand, not considering the ambient air chemical composition may introduce environmental interferences, while, in parallel, subtracting air levels from breath may result in the exclusion of valuable information.

Some further issues should be considered when interpreting the results of the present study. Although SPME has many advantages as a solvent-free and versatile pre-concentration method, it is not without limitations. During SPME, VOCs compete for the active sites of the fiber, and molecules with higher molecular weight may displace smaller ones. Thus, varying the composition of samples may influence the amounts of VOC extracted [48]. Moreover, different fiber coatings are suitable for different classes of analytes [49]. The fiber used in this study (CAR/PDMS) is suitable for VOCs with low molecular weight and a Kovats index of less than 980 [50]. According to a study conducted to evaluate the performance of different fiber coatings in the isolation of VOCs from feces, the particular fiber used isolated 60% of the total examined VOCs [51]. Concerning sampling, pre-concentration and instrumental procedures, we adopted a mixed expiratory breath sampling/SPME/GC–MS approach, but a variety of alternative methods are available. In brief, sampling can also focus on later or end-tidal expiratory breath, pre-concentration can be achieved with thermal desorption (TD) and needle trap devices (NTDs) [52] and instrumental analysis can also be performed with proton transfer reaction MS (PTR-MS) and selective ion flow tube MS (SIFT-MS) [18]. Cross-reactive sensors have also been developed and tested by numerous research groups [53].

Another limitation of this study is that the participants who formed the HC group did not undergo clinical examination or diagnostic tests to exclude the possibility of having undiagnosed cancer or serious pulmonary diseases, instead they were recruited based on personal interviews. Thus, the possibility that a few individuals were falsely classified as controls cannot be entirely excluded.

In summary, untargeted VOC profiling captured, to a large extent, the information provided by targeted analysis and performed more efficiently in discriminating lung cancer patients from patients with benign pulmonary diseases, through the utilization of new compounds that were not previously considered. However, uncertainties in compound identification and automated processing of raw data should be carefully addressed. Subsequence steps for the verification and manual correction of automatically identified peaks in the raw chromatographic files can increase the reliability of the acquired datasets.

## 4. Materials and Methods

### 4.1. Participant Recruitment and Breath Sampling

A detailed description concerning the procedures followed for participant recruitment and sampling of exhaled breath can be found in a previous publication [10]. In brief, the study population consisted of 85 patients from the General University Hospital of Larissa (Greece) who underwent bronchoscopy due to abnormal CT findings and a control group of 52 individuals of similar age were recruited from local health centers. Samples were collected from October 2018 to October 2019. After bronchoscopy, patients were categorized according to the presence of LC, according to results of the cytological/histological examination. The control group (referred to in the text as healthy controls (HC)) was selected on the basis of the absence of self-reported pulmonary diseases and cancer. The absence of these diseases was determined by self-report during the personal interviews conducted on the day of sampling.

Breath samples were collected in Tedlar^®^ bags (Sigma-Aldrich, St. Louis, MO, USA). Participants were asked to inhale deeply and hold their breath for 30 s, then exhale through a disposable mouthpiece into the 1 L Tedlar^®^ bag until filled. Two breath samples were collected with approximately two-minute intervals in between. Ambient air samples were also collected with the use of a portable Laboport^®^ UN 86 KTP (KNF Neuberger GmbH, Freiburg, Germany) pump.

### 4.2. Materials, Solid Phase Microextraction and GC–MS Analysis

A detailed description of the materials and methods used in the present study can be found in our previous publication [10]. In brief, extraction and pre-concentration of the analytes from breath samples was achieved by solid phase microextraction (SPME) using a 75 μm carboxen-polydimethylsiloxane (CAR/PDMS)-coated fused silica fiber assembly (Sigma-Aldrich, St. Louis, MO, USA), and desorption of analytes from the fiber was performed for 5 min at 270 °C. Instrumental analysis was performed with a Finnigan Trace GC Ultra/Polaris Ion Trap GC/MSn system equipped with a DB-624 GC capillary column (inner diameter: 0.25 mm, length: 30 m, film: 1.4 μm, 6% cyanopropylphenyl/94% dimethylpolysiloxan, Agilent, Santa Clara, CA, USA). GC–MS chromatograms were acquired in total ion current (TIC) mode of the mass analyzer, and then extracted at one or two specific *m*/*z* values for analyte quantification. Data acquisition and processing were carried out using Xcalibur™ 3.0 software (ThermoFisher Scientific, San Francisco, CA, USA). Furthermore, for the determination of RIs, SAK-100-1 and SMA-200-1 (Agilent, Santa Clara, CA, USA) analytical standards containing C5 to C12 alkanes were used. Gas samples were prepared, spiked with methanolic solution of C5-C12 alkanes and retention times of each alkane were determined.

### 4.3. Data Pre-Processing and Analysis

After GC/MS analysis, all raw data were converted to mzml files using ProteoWizard, and subsequently the converted files were imported into XCMS Online software (XCMS Online version 3.7.1) (https://xcmsonline.scripps.edu) for feature detection, alignment and retention time correction. The raw data processing was carried out using the following parameters: general: Rt, format: minutes, polarity: positive, feature detection: centWave, ppm: 900, minimum peak width: 5, maximum peak width: 30, mzdiff: 0.1, signal/noise threshold: 3, integration method: 1, prefilter peaks: 3, prefilter intensity: 100, noise filter: 0, Rt. correction: obiwarp, profStep: 1, alignment: bw 1, minfrac: 0.2, mzwid: 0.25, minsamp: 1, max: 500, statistics: statistical test: *t*-test. All chromatograms were simultaneously analyzed with identical settings. Selection of the most informative variables (*m*/*z*) was based on statistical criteria (*p*-value < 0.01, fold change > 2, *m*/*z* < 140, Rt < 14.00 min for Ca+ vs. HC; *p*-value < 0.05, fold change > 1.1, *m*/*z* < 140, Rt < 14.00 min for Ca+ vs. Ca−) of differentiated peak intensity between patients and controls.

### 4.4. Identification of Candidate Features and Raw Data Reprocessing

All features identified as differentiated between population groups with the XCMS analysis were searched for in the raw chromatograms and the corresponding peaks were identified. The mass spectrum of the identified peaks was studied in comparison with the National Institute of Standards and Technology (NIST) spectrometric library. Peaks of compounds corresponding to technical interferences (siloxanes, Tedlar^®^ bag compounds) were excluded from further analysis. Extracted ion chromatograms were obtained for the ions identified as significantly differentiated between population subgroups by XCMS analysis, and were reprocessed by calculation of the areas of the chromatographic peaks in SIM mode using Thermo Xcalibur™ software. The most discriminatory features were assigned based on mass spectral similarities to the NIST 2011 mass spectral library. Compounds were categorized as “probable” (probability > 75%), “possible” (probability 50–75%) and unknown (probability < 50%). To further confirm the identification of compounds, retention characteristics were examined. Retention times were simulated by using the Pro EZGC Chromatogram Modeler (Restek Corporation, Bellefonte, PA, USA), introducing an equivalent chromatographic column and an identical temperature program. Simulated RTs were compared to actual RTs for substances contained in the Restek database. Retention indices of these compounds were retrieved from the NIST webbook and related to a fully non-polar column (100% polydimethylsiloxane). Moreover, retention indices for each compound were experimentally determined. SAK-100-1 and SMA-200-1 (Agilent) analytical standards with C5 to C12 alkanes were used to calculate the retention indices from the unknown compounds. Experimental retention indices of these compounds were calculated according to the following formula:

I = 100 [n + (t_i_ − t_n)_/(t_n+1_ − t_n)_]

I: retention index
n: number of carbons of heading *n*-alkane peak *i*
t_i_: retention time of specific compound *i* (minutes)
t_n_, t_n+1_: retention times of heading and trailing n-alkanes

Normalization of chromatographic peak areas was performed with an external standard, by dividing instrument response by the geometric mean peak areas of three monoaromatic compounds (benzene, toluene and ethyl benzene) of a standard mixture (≈20 ng/L air each) analyzed on the same day.

### 4.5. Machine Learning Methods

The machine learning analyses were performed with Waikato Environment for Knowledge Analysis (Weka). For each comparison, group 1 vs. group 2 or cases vs. controls were analyzed using naive Bayes, logistic regression and random forest methods, with 10-fold cross-validation. However, random forests consistently outperformed the other algorithms, therefore, all results are shown for this specific type of algorithm. Feature selection within the appropriate Weka module was also performed, in order to detect subsets of informative metabolites that could more efficiently separate the groups from each other. In particular, feature selection was performed in two steps with a wrapper that evaluates various subsets of the features (WrapperSubsetEval), using the Best_First method in order to maximize the performance of the random forest, based on the metric of the area under the curve (AUC). In the first step, the wrapper functions in a feature selection mode that performs 10-fold cross-validation. The output of this first feature selection step assesses how many times a feature has been selected in the 10-fold cross-validations. The features that are selected in at least 50% of the cross-validations form another subset that is fed into the second step. Thus, we repeat (in the second step) the feature selection, by starting with the abovementioned informative subset, and this time the wrapper runs in a feature selection mode that uses the full training set and selects only a certain final subset of features.

## Figures and Tables

**Table 1:** Identification of compounds based on spectra comparison with NIST library and retention time criteria.

Candidate Compound	Probability (NIST), %	Match Score (NIST)	Retention Time, min	Retention Time Simulated ^1^, min	Deviations in Retention Time, %	Experimentally Determined Retention Index	NIST Retention Index ^2^	Deviations in Retention Index, %
3-methyl-furane	86	892	5.03	NA		615	602	2.16
acetaldoxime	53	753	5.38	NA		625	606	3.14
Benzene *	72	923	7.17	7.73	−7.81	677	647	4.64
acetic acid	59	912	7.86	NA		698	650	7.38
1-methoxy-2-propanol	69	891	8.27	7.83	5.32	711	658	8.05
dimethyl furane	78	852	8.33	8.66	−3.96	714	694	2.88
methyl propyl sulfide	89	840	8.79	NA		729	714	2.10
1-methylthio-(E)-1-propene **	90	877	9.57	NA		756	722	4.71
Toluene *	34	868	10.31	10.95	−6.21	782	750	4.27
propionic acid	78	702	10.59	NA		792	712	11.24
p- xylene **	81	845	12.00	12.1	−0.83	859	833	3.12
ethyl benzene *	61	877	12.10	12.38	−2.31	890	858	3.73
Styrene *	37	869	12.3	12.79	−3.98	906	876	3.42
methylacetamide	78	831	12.79	NA		959	825	16.24
p-benzoquinone	90	817	13.00	NA		982	888	10.59
N-2-Aminoethyl acetamide	62	800	13.2	NA		1005	NA	
eucalyptol	52	848	13.51	NA		1060	1017	4.23

* Verified by analytical standard. ** NIST probability is given for all isomer compounds. Mass spectra were very similar for isomers of these compounds, compounds were identified based on RI similarities. ^1^ Retention time was simulated with Pro EZGC Chromatogram Modeler, Restek Corporation. ^2^ Retention indices were derived from NIST database related to a fully non-polar column (100% polydimethylsiloxane). NA: not available with equivalent column.

**Table 2:** Comparative analysis of the areas of the 29 chromatographic peaks between patient groups and relative presence in ambient air.

Compound	Relative Presence in Ambient Air ^1^	Ca+/HC		Ca+/Ca−	
		Trend in LC Patients	Significance *	Trend in LC Patients	Significance *
unknown	insignificant	↑	0.052	↑	0.311
unknown	moderate	↓	0.071	↓	0.056
3-methyl-furan *	low	↓	0.514	↓	0.482
acetaldoxime	high	↓↓↓	<0.001	↓	0.341
unknown	moderate	↓↓↓	<0.001	↓	0.689
unknown	low	↑↑	0.01	↓↓	0.013
benzene	moderate	↓↓↓	<0.001	↓	0.089
unknown	moderate	↓↓↓	<0.001	↓	0.756
acetic acid	low	↓↓↓	<0.001	↓	0.979
1-methoxy-2-propanol	high	↓↓↓	<0.001	↓	0.272
dimethyl furan	low	↓	0.125	↓	0.286
unknown	moderate	↑↑↑	0.002	↓	0.396
unknown	moderate	↓	0.902	↓	0.082
methyl propyl sulfide	insignificant	↓↓↓	<0.001	↓↓	0.035
1-methylthio-(E)-1-propene	insignificant	↓↓↓	<0.001	↓	0.239
unknown	insignificant	↓↓↓	<0.001	↓	0.185
toluene	moderate	↑↑↑	0.001	↑	0.986
propionic acid	insignificant	↓↓↓	<0.001	↑	0.384
unknown	high	↑	0.053	↑	0.752
unknown	moderate	↓	0.124	↓	0.175
ethylbenzene	moderate	↑↑↑	<0.001	↑	0.618
xylene(p,o,m)	moderate	↑↑↑	<0.001	↑	0.434
styrene	moderate	↑↑↑	<0.001	↑	0.423
methylacetamide	high	↓	0.178	↑	0.539
p-benzoquinone	insignificant	↓	0.076	↓	0.388
N-2-Aminoethyl acetamide	moderate	↓↓↓	<0.001	↓	0.824
unknown	moderate	↓↓↓	<0.001	↓	0.104
eucalyptol	low	↑	0.066	↑	0.511
unknown	moderate	↑	0.092	↑	0.463

^1^ Determined from mean breath/mean air ratio. Insignificant: >20, low: 5–20, moderate: 0.5–5, high: <0.5. * Significance determined by Mann–Whitney test. ↑, ↓: *p* > 0.05, ↑↑, ↓↓: *p* = 0.01–0.05, ↓↓↓, ↑↑↑: *p* < 0.01.

**Table 3:** Results of machine learning methods (random forest) to estimate the discrimination efficiency of the breath analysis.

Analysis no.	Approach	Variable	Comparison Groups	Smoking Habit	Features Used	Accuracy	AUC
1	targeted	Br	Ca+ vs. HC	All	t1–t19	85.14	0.95
2	targeted	Br	Ca+ vs. HC	All	t4, t5, t7–t11,t13–t15,t18	89.10	0.97
3	targeted	Br	Ca− vs. HC	All	t1–t19	86.36	0.91
4	targeted	Br	Ca− vs. HC	All	t4,t5, t7–t17	88.63	0.94
5	targeted	Br	Ca+ & Ca− vs. HC	All	t1–t19	86.70	0.96
6	targeted	Br	Ca+ & Ca− vs. HC	All	t1, t4,t5,t7–t15,t17	90.50	0.96
7	targeted	Br	Ca+ vs. Ca−	All	t1–t19	43.50	0.39
8	targeted	Br	Ca+ vs. Ca−	All	t4,t9, t17	52.90	0.55
9	untargeted	Br	Ca+ vs. HC	All	u1–u29	86.14	0.94
10	untargeted	Br	Ca+ vs. HC	All	u4,u8,u12,u14,u16,u19,u28,u29	91.08	0.96
11	untargeted	Br	Ca− vs. HC	All	u1–u29	89.77	0.94
12	untargeted	Br	Ca− vs. HC	All	u4,u6, u8, u12,u26,u27,u29	94.3	0.97
13	untargeted	Br	Ca+ & Ca− vs. HC	All	u1–u29	86.9	0.95
14	untargeted	Br	Ca+ & Ca− vs. HC	All	u4, u8, u11,u12,u19,u22,u26,u27, u29	92	0.97
15	untargeted	Br	Ca+ vs. Ca−	All	u1–u29	52.9	0.54
16	untargeted	Br	Ca+ vs. Ca−	All	u4, u20,u26	75.3	0.82
17	untargeted	Sbtr	Ca+ vs. Ca−	All	t1–t19, u1–u29	57.6	0.54
18	untargeted	Sbtr	Ca+ vs. Ca−	All	u2, u4,u6, u11,u14, u25, u28,u29	71.76	0.78
19	merged	Br	Ca+ vs. Ca−	All	u1–u29, t1–t19	44.7	0.44
20	merged	Br	Ca+ vs. Ca−	All	t9, u4, u26	72.9	0.72
21	untargeted	Br	Ca+ vs. Ca−	Non-smokers	u1–u29	59.4	0.57
22	untargeted	Br	Ca+ vs. Ca−	Non-smokers	u4, u20,u 26	72.5	0.68
23	untargeted	Br	Ca+ vs. Ca−	Non-smokers	u4, u11, u13,u20,u26	76.8	0.85

Br: corresponds to breath compound levels, Sbtr: corresponds to breath subtract levels, Ca+: patients diagnosed with lung cancer, Ca−: patients with pathological CT findings not diagnosed with lung cancer by histological/cytological examination, HC: healthy controls. Features from targeted analysis: t1: isoprene, t2: acetone, t3: 2-propanol, t4: hexane, t5: 1-propanol, t6: 2-butanone, t7: cyclohexane, t8: benzene, t9: thiophene, t10: 1-butanol, t11: toluene, t12: octane, t13: ethyl butyrate, t14: hexanal, t15: ethyl benzene, t16: styrene, t17: cyclohexanone, t18: octanal, t19: nonanal. Features from untargeted analysis: u1: unknown, u2: unknown, u3: 3-methyl-furan, u4: acetaldoxime, u5: unknown, u6: unknown, u7: benzene, u8: unknown, u9: acetic acid, u10: 1-methoxy-2-propanol, u11: dimethyl furan, u12: unknown, u13: unknown, u14: 1-methylthio-(*E*)-1-propene, u15: allyl methyl sulfide, u16: unknown, u17: toluene, u18: propionic acid, u19: unknown, u20: unknown, u21: ethylbenzene, u22: p-xylene, u23: styrene, u24: methylacetamide, u25: p-benzoquinone, u26: N-2-aminoacetyl acetamide, u27: unknown, u28: eucalyptol, u29: unknown.

## Data Availability

The data are not publicly available because they contain sensitive information at an individual level.

## Sample Availability

Air samples cannot be stored and thus they are not available.
